# Exploring the Infiltration Features of Perovskite within Mesoporous Carbon Stack Solar Cells Using Broad Beam Ion Milling

**DOI:** 10.3390/ma14195852

**Published:** 2021-10-06

**Authors:** Tom Dunlop, Owen Kesteven, Francesca De Rossi, Pete Davies, Trystan Watson, Cecile Charbonneau

**Affiliations:** 1 SPECIFIC IKC, Faculty of Science and Engineering, Bay Campus, Swansea University, Fabian Way, Swansea SA1 8EN, UK; 707096@swansea.ac.uk (O.K.); effe.derossi@gmail.com (F.D.R.); t.m.watson@swansea.ac.uk (T.W.); c.m.e.charbonneau@Swansea.ac.uk (C.C.); 2 AIM, Faculty of Science and Engineering, Bay Campus, Swansea University, Fabian Way, Swansea SA1 8EN, UK; p.davies@swansea.ac.uk

**Keywords:** broad beam ion milling, ion milling, perovskite, SEM, EDS, mesoporous carbon stack, mesoscopic perovskite solar cell, infiltration

## Abstract

Carbon perovskite solar cells (C-PSCs) are a popular photovoltaic technology currently undergoing extensive development on the global research scene. Whilst their record efficiency now rivals that of silicon PV in small-scale devices, C-PSCs still require considerable development to progress to a commercial-scale product. This study is the first of its kind to use broad beam ion milling for C-PSCs. It investigates how the carbon ink, usually optimised for maximum sheet conductivity, impacts the infiltration of the perovskite into the active layers, which in turn impacts the performance of the cells. Through the use of secondary electron microscopy with energy-dispersive X-ray spectroscopy, infiltration defects were revealed relating to carbon flake orientation. The cross sections imaged showed between a 2% and 100% inactive area within the C-PSCs due to this carbon blocking effect. The impact of these defects on the performance of solar cells is considerable, and by better understanding these defects devices can be improved for mass manufacture.

## 1. Introduction

After a decade of research, perovskite photovoltaics are now certified with power conversion efficiencies (PCEs) of up to 25.2% at the laboratory scale and are starting to make their appearance in large manufacturing facilities [1,2,3]. A multitude of architectures and materials have been developed along the way, including the carbon-based mesoporous triple stack. This technology, first introduced by Ku et al. [4], relies on the sequential deposition of mesoporous to macroporous TiO_2_, ZrO_2_, and carbon layers, on top of a transparent conductive substrate, usually Fluorine doped Tin Oxide (FTO)-coated glass. The pores of the stacked layers are then infiltrated with a wet lead halide organic precursor which crystallises into a perovskite light-absorbing material. This type of perovskite cell offers advantages in terms of materials costs and processing [5,6,7]. For instance, the carbon ink used to prepare the hole transport layer is more cost effective than spiro-MeOTAD, a semiconductor organic material frequently used in small quantities in research laboratories [8,9]. Despite this, there is no need to compromise significantly on efficiency, given that a recent record of 17% PCE has been reported by Liu et al. [9]. This architecture has also been shown to be stable, with proven year-long energy outputs [10]. From the manufacturing point of view, the materials deposition is compatible with already well-established printing equipment, namely screen-printing equipment, enabling continuous low-cost processing at high production rates [11]. Recent work by De Rossi et al. [12,13] demonstrated the successful fabrication of modules of over 198 cm^2^ with PCEs of over 10%, where screen printing was central to their manufacture. In parallel, other technical advances have enabled the automation of the infiltration step via a transfer from manual pipetting [14] to inkjet printing [15] and mechanised mesh infiltration [16]. These developments show that there is a bright commercial future ahead for C-PSCs, and this is supported by a growing interest within the global scientific community, with many research groups developing this technology.

However, to achieve the performance and manufacturability of current photovoltaic (PV) market-leading technologies such as c-Si, CIGS, or CdTe, carbon-based perovskite solar cells still need to overcome a few hurdles. These are typically concerned with improving the reproducibility of device performance, which is anticipated to be associated with structural defects. Previous investigations by Lakhiani et al. [17], undertaken using combined high-resolution multi-modal mapping techniques demonstrated the occurrence of voids, e.g., the absence of crystallised perovskite material within the m-ZrO_2_ and m-TiO_2_ layers. Baker et al. [18] investigated the wetting of the precursor as a possible cause of these, but found that all porous materials used in the device had excellent wetting properties (complete infiltration < 0.2 s). Chen et al., also studied the wetting of the perovskite precursor on the carbon layer and attributed variations in device performance to infiltration yields, but no observable evidence of pore filling was shown [19]. Finally, methylammonium lead iodide (MAPI) with 5-AVAI additions was shown in previous works to provide the best infiltration and stability among the MAPI variants, for large-scale devices [20,21,22,23].

**Figure 1:**
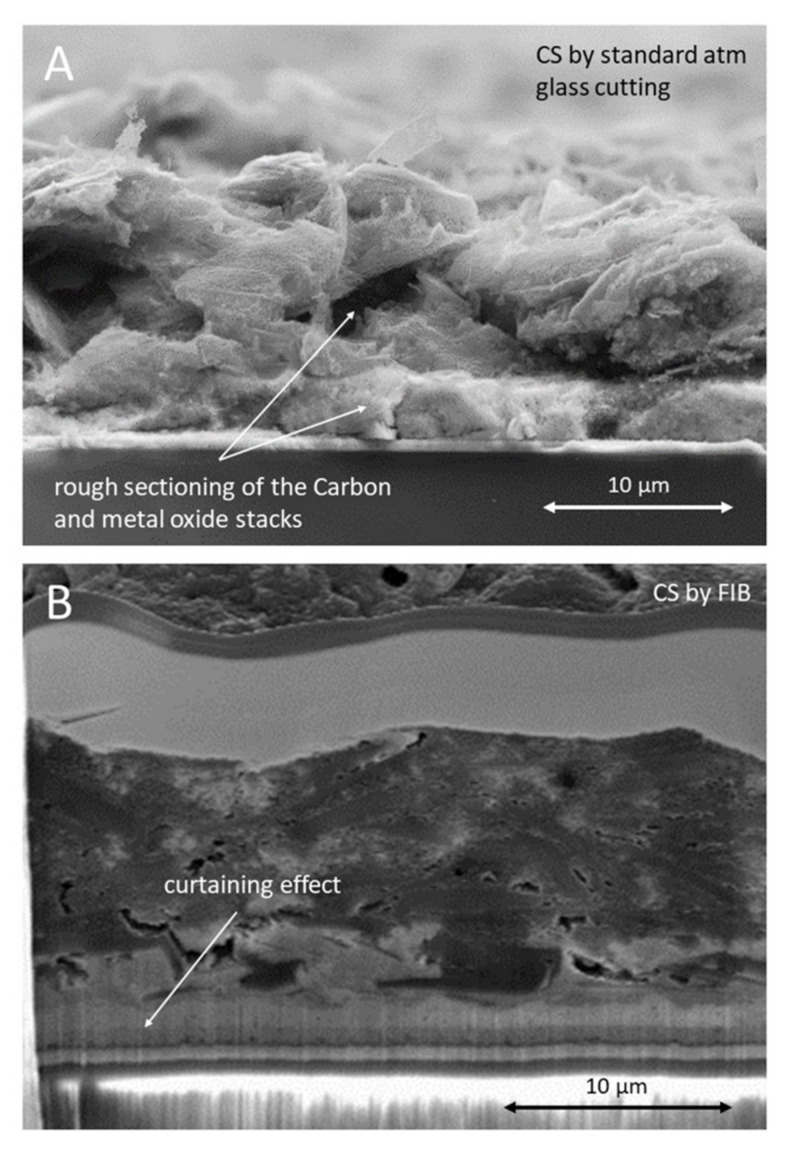
SEM observation of cross sections of the carbon triple stack prepared by application of: (**A**) atmospheric glass cutting vs. (**B**) focused ion beam milling. The procedures are briefly described in Section 2.

There are currently no studies examining the impact of the morphological features characteristic of the triple stack (C, ZrO_2_, and TiO_2_) on the pore filling of the infiltered perovskite. This is mainly due to difficulties in the preparation of perovskite samples for cross-section observations at high magnification. Traditionally, a standard fracture method such as cutting samples in the ambient environment or in nitrogen is adequate for imaging planar cells and has been used for sample preparation [24,25]. This is a very simple technique, involving the scribing of a trench at the bottom of the glass substrate followed by fracturing using compressive forces on either side of the mechanically weakened area. It is commonly applied for preparing cross sections of simple layered structures and enables good-quality high-magnification observations when the stack is thin (<1 micron), for instance in planar perovskite photovoltaic devices [26]. However, this technique is not well adapted to preparing cross sections of C-PSC devices, which are characterised by a much greater overall thickness (approximately 10–12 microns) and materials of variable hardness and density across the stack (with a Young’s modulus of 19–157 GPa between the layers [27,28,29]). Typically, the application of this method leads to the production of rough sections, as shown in Figure 1A. A high surface roughness can also generate steps, hiding information on the infiltration of the perovskite in areas of interest [30]. This also affects the quality of the EDS analysis and the chemical mapping of materials [31,32,33]. In addition, carbon flakes typically fail to fracture with the rest of the stack. Due to their large dimensions, they have been observed protruding from, or hanging over, the layers of interest, making any form of characterisation impossible [34,35]. Standard fracture techniques can be used in cryogenic conditions to improve the brittleness of the stack, using liquid N_2_ prior to fracture. However, once fractured, the cross section is rapidly subjected to localised condensation of moisture, causing the perovskite material to degrade almost instantly to PbI_2_ [36].

Two ion beam milling techniques, namely, focused ion beam (FIB) and broad beam ion milling (BBIM), have been shown to provide an improved cross section [30,37,38]. Ion beam sources are typically either argon or gallium. FIB operates within a scanning electron microscope (SEM) and can be used for selective material deposition [39], local milling and polishing for TEM observations [40], and microfabrication of devices [41].

Ion milling techniques offer a unique opportunity to prepare high-quality cross sections and have been used in many application fields [38,42]. FIB was not investigated in detail in this study due to the long time required to produce a very small cross section with a number of surface defects such as curtaining and redeposition. An example of this can be seen in Figure 1B. In addition, lead halide organic perovskites are prone to decomposition when exposed to excessive temperatures and reactive metals. Artefacts observed when ion beam milling include the following. (1) Curtaining, characterised by the appearance of lines and irregular surfaces at the milled faces. Curtaining is caused by spatial variation in the sputtering rate of the sample and the changes in the current density caused by the ion scattering. It is particularly problematic in porous materials and composites of hard and soft materials, such as the triple stack. (2) Rippling, where self-organisation occurs because of the process instability and the large ion size compared with those in the solid [30] (1.36 Å for Ga and 1.08 Å for Xe vs. 0.71 Å for Argon). (3) Crystal lattice damage due to gallium and xenon ions in particular [43], which cause localised melting that modifies the structure and introduces point defects. The size of these artefacts may be less of an issue compared to the scale of the perovskite infiltration features studied here but should not be neglected. (4) Finally, the use of a gallium source is not recommended, as gallium atoms can infiltrate materials to such an extent that local concentrations can affect the quantitative elemental analysis [40]. Through this process, gallium is also known to weaken the grain boundaries of crystalline materials. This is of particular concern in the case of lead halide perovskite materials, which are naturally prone to degrading via localised halide ion segregation at the grain boundaries [44].

BBIM systems enable the polishing of much larger areas than FIB techniques, and therefore they are used widely for sample preparation for electron backscatter diffraction (EBSD). More specialised uses can include the cross-sectioning of materials, and recent studies have even developed serial section tomography allowing the characterisation of 3D materials [38]. Initial work carried out by Lakhiani et al., showed the correlation between top-view multi-modal microscopy, combining photoluminescence, electroluminescence, photocurrent, and Raman signal techniques, with structural defects observed in BBIM-polished cross sections of the same samples [17]. Here, we develop this method further by systematically determining the optimum settings for the BBIM preparation of clean C-PSC cross sections. These settings are then applied to the preparation of C-PSC devices with different performances, to support a very precise investigation of the perovskite infiltration features through all the layers of the porous triple stack. By combining SEM observations with electron-dispersive spectroscopy (EDS), we demonstrate the occurrence of different types of defects resulting primarily from the orientation of the screen-printed carbon flakes, such as voids, inclusions, and areas characterised by low levels of infiltration. This study supports the fundamental understanding of how the perovskite precursor infiltrates the mesoporous carbon stack and informs research teams working on the manufacturing of large-scale C-PSC devices regarding how structural and print optimisation of the C layer may enable this technology to achieve higher manufacturing reproducibility and increased outputs.

## 2. Materials and Methods

### 2.1. Fabrication of the C-PSC Triple Stack

TEC 7 fluorine-doped tin oxide (FTO) glass substrates were etched using a Rofin Nd:YVO4 laser (Rofin, Plymouth, MI, USA) with a current of 32A, a frequency of 4700 Hz pulsing at 10 µs, and a speed of 150 mms^−1^. The substrates were rinsed in isopropanol and di-ionised water before being O2 plasma treated. A compact TiO_2_ layer (50 nm) was deposited via spray pyrolysis at 300 °C from a solution of 1/9 *v*/*v* of titanium diisopropoxide bis(acetylacetonate), 75 wt.% in isopropanol. Dilute 30 NR-D TiO_2_ paste (2/3 *w*/*w* paste/terpineol, 30 nm particle size, Dyesol, West Perth, WA, Australia) was screen printed onto the compact TiO_2_ and then sintered at 550 °C for 30 min on a hotplate. Zr-Nanoxide ZT/SP paste (1/5 *w*/*w* particle/terpineol, 20–40 nm particle size, Solaronix, Aubonne, Switzerland) was screen printed overlapping the TiO_2_ and sintered at 400 °C for 30 min. Finally, a carbon paste (C2150317D3, Gwent Electronic Materials, Pontypool, UK) was screen printed onto the zirconia layer and sintered at 400 °C for 30 min on a hotplate. The carbon paste contained flakes with an average size of 22 μm and a size range of 5–50 μm, and nanoparticulate carbon black.

The perovskite precursor 5-AVAI-MAPbI3 (3% n/n 5-AVAI/MAI) was prepared by dissolving PbI_2_ (99.99%, TCI chemicals, Oxford, UK) and methyl ammonium iodide (MAI, Dyesol, West Perth, WA, Australia) in γ-butyrolactone (GBL, 99%, Sigma-Aldrich, St Louis, MO, USA) solvent under stirring at 70 °C to reach a concentration of 0.952 M. The solution was drop casted onto the dry triple stack at ambient temperature (~21 °C) and left to dry and settle for 10 min before being annealed in a fan oven at 50 °C for 1 h [11].

### 2.2. Cross-Sectioning Techniques

#### 2.2.1. Glass Cutting

Samples were scribed using a diamond scribe on the clean side of the glass opposite to the printed films and fractured by hand. This provided the cut edge for the standard fracturing as shown in Figure 1 and also the starting point for BBIM cross-sectioning.

#### 2.2.2. Focused Ion Beam

Focused ion beam milling was undertaken using a Zeiss Crossbeam 550 FIB-SEM (Carl Zeiss AG, Oberkochen, Germany). The cross section to be imaged was prepared by first depositing a Pt protection layer (30 kV 100 pA IB probe) and then milling a wedge-shaped trench into the surface, initially using a 30 kV, 7 nA FIB probe. Milling was then performed with progressively lower powers in order to improve the finish (30 kV 3nA, 30 kV 1.5 nA, 30 kV 700 pA, and 30 kV 300 pA).

#### 2.2.3. Broad Beam Ion Milling

Samples were milled in a Hitachi IM4000 broad beam argon ion miller (Hitachi, Tokyo, Japan). Sections of cells with areas of 1 cm^2^ were milled, using voltages between 3 kV and 5 kV over durations of of 1 h to 4.5 h. A slow C3 stage rotation was used to ensure an evenly milled surface, with an argon gas flow of 0.07 cm^3^/min used throughout.

### 2.3. Electron Microscopy

Samples were mounted onto a conductive holder and coated with 5 nm Pt to provide conductivity for the glass substrate. Imaging was undertaken on a JEOL JSM-7800F field emission SEM (JEOL, Tokyo, Japan) with an electron energy of 20 kV and current density of 200 pA. Images were acquired in secondary electron scanning mode. The chemical analyses of the cross sections were performed using an Oxford Instruments Ultim energy-dispersive X-ray spectroscopy (EDS) detector with an AZTEC software (Ver 5.0) analysis package (Oxford Instruments Plc, Abingdon, UK), at a 10 mm working distance with the samples angled towards the detector to improve the signal.

## 3. Results and Discussion

### 3.1. Optimisation of Broad Beam Ion Milling Operating Parameters

**Figure 2:**
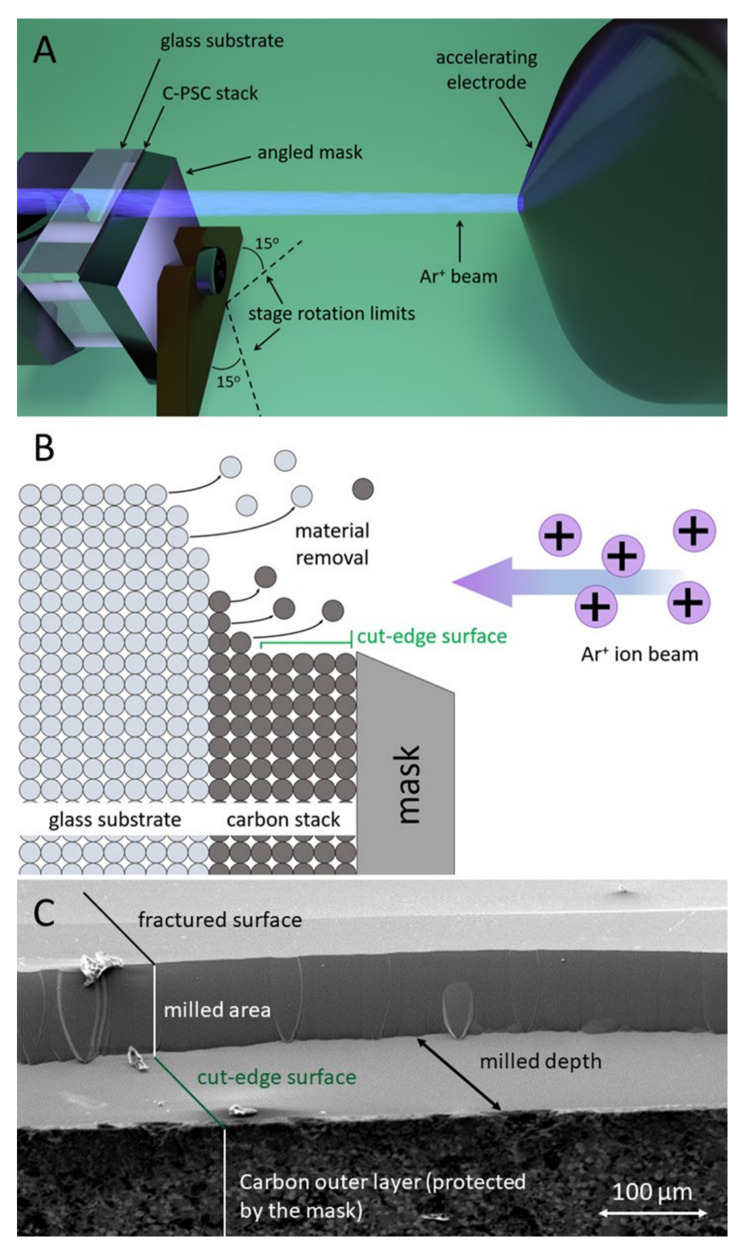
(**A**) Representation of sample positioning and ion source inside the BBIM chamber; (**B**) side view schematic representing the milling of the sample; (**C**) top view SEM observation of the sample post BBIM (4 kV for 1.5 h).

In this study, the cross-sectioning of the C-PSC stacks was performed as illustrated in Figure 2. The sample was positioned perpendicularly to the Ar ion beam source (Figure 2A). A metal mask was placed on top of the C-PSC stack with its edge aligned just below the level of the sample fracture. This enabled the selective removal of material between the rough fractured edge of the sample and the edge of the mask, leading to the formation of a straight section through the thickness of the C-PSC stack and the glass substrate, labelled the cut-edge surface (Figure 2B). A rotation of the sample stage (from −15° to +15° from the direction of the ion beam source) was applied during the milling operation, to limit the appearance of curtaining artefacts caused by the ion beam. The resulting mill shape and area of removal are shown in Figure 2C. After milling, the C-PSC stack presented a straight section whilst the area removed above the mask was characterised by a curved base, owing to the Gaussian shape of the ion beam.

**Figure 3:**
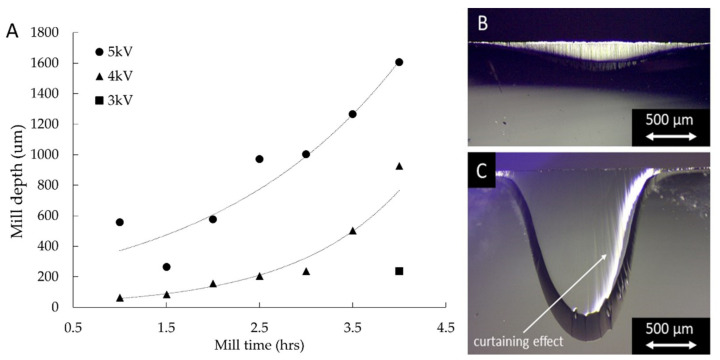
(**A**) Effect of ion beam source accelerating voltage and milling time vs. mill depth. The inserts show optical microscope images of the side view of a sample milled (**B**) at 4 kV for 1.5 h and (**C**) at 5 kV for 3.5 h (both are reported in (**A**)).

In order to ensure the optimum milling parameters for the cross sections of this layered structure, the accelerating voltages and milling times were varied. Figure 3A shows the relationship between the ion source energy (in kV), the milling time and the depth. The mill was considered successful when it was characterised by a depth of at least 100 μm, enabling good quality cross-sectioning of the sample through the entire thickness of the C-PSC stack (approximately 20 µm) and glass substrate, over a length of 1–2 mm, as shown in Figure 3B. With an accelerating voltage lower than 3 kV, a milling time of over 3 h is required to achieve this, which is an operating time comparable to that typically required for preparing cross sections using in situ focused ion beam milling, and hence is considered excessive. By increasing the energy of the ion source to 4 and 5 kV, the milling time required to obtain a mill depth of approximately 100 μm is successfully reduced to below 1.5 hrs. However, optical microscopy observations of the samples obtained by application of a 5 kV ion beam source revealed evidence of damage, including excessive curtaining, as shown in Figure 3C. Therefore, an accelerating voltage of 4 kV provides the best compromise between the time required and the mill quality. This is illustrated in Figure 3B, where a maximum mill depth of ~200 µm is obtained after 1.5 h of milling at 4 kV, resulting in the provision of a clean C-PSC cross section over ~1.5 mm. Milling times longer than 2 h were observed to cause excessive heating of the sample, damaging the perovskite material.

### 3.2. Features within the Perovskite Carbon Stack

#### 3.2.1. Structure of the Mesoporous Stack

**Figure 4:**
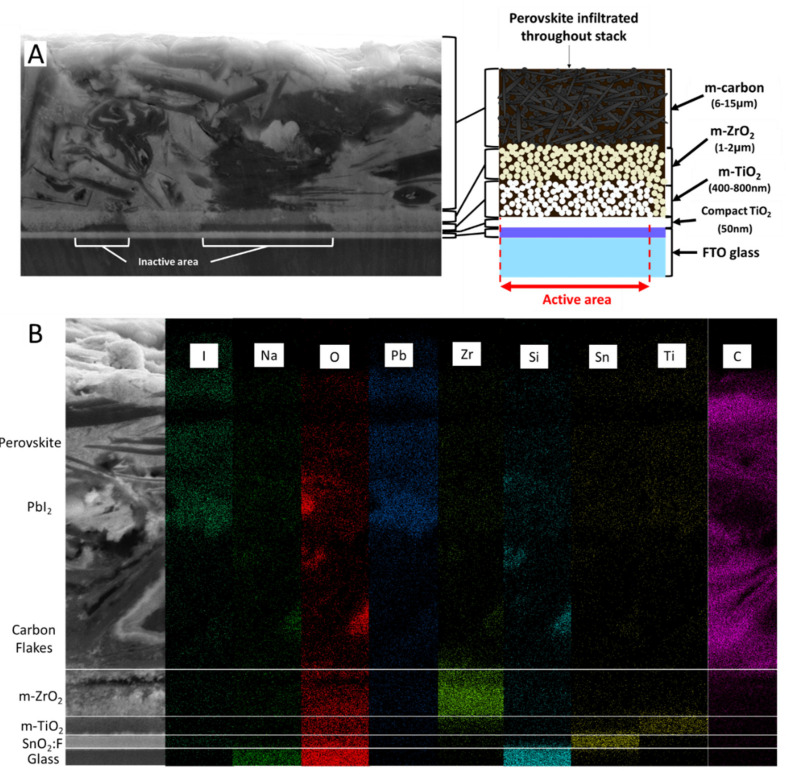
(**A**) Cross section and schematic of the full perovskite carbon stack with inactive areas identified. (**B**) EDS pattern of the triple stack clearly showing the printed layers.

The PCS cross sections were prepared using the optimised broad beam milling parameters of 4 kV over 1.5 h. The resulting sections were investigated using secondary electron imaging (Figure 4A) and energy-dispersive X-ray elemental analysis (Figure 4B), a combination which reveals the infiltration behaviour of MAPI within the carbon stack solar cells. Figure 4 clearly shows the stacking of all layers, starting with the glass substrate at the base which is coated with a compact layer of approximately 540 nm of fluorine-doped tin oxide (FTO), followed by 50 nm of compact TiO_2_ (deposited by the spray pyrolysis of TiAcAc), two mesoporous layers of TiO_2_ (650 nm thickness) and ZrO_2_ (1 μm thickness) nanoparticles, and finally a 20 μm porous layer of carbon on top. All the porous layers were produced by screen printing and annealing at a high temperature. The perovskite material precursor is infiltrated as a wet solution from the top of the stack and cascades down all the way to the compact layer of TiO_2_. The intricate structure of the stack, occasional manufacturing defects, and variations in the wetting properties of the wet precursor with the various materials, make the full infiltration of the stack a challenging process. The use of AVAI has been demonstrated by Lakhiani et al. [17] to mitigate these difficulties; however, there is still room for improvement.

#### 3.2.2. By-Products, Infiltration, and Structural Defects within the Carbon Layer

**Figure 5:**
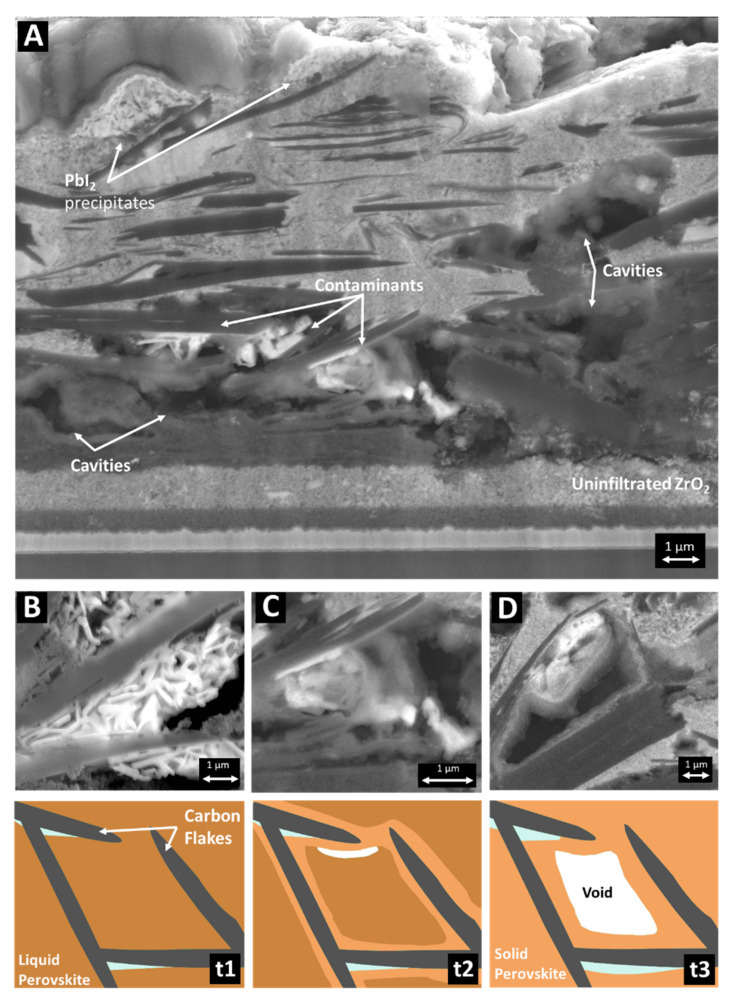
(**A**) An example of a poorly infiltrated carbon stack with key defects identified; (**B**) residual PbI2 precipitates; (**C**) contaminants (Pb free); (**D**) infiltration cavity with some retained PbI2. In t1–t3, schematics of MAPI in DMSO (brown) infiltrated into mesoporous carbon (grey) are shown: t1, fresh infiltration; t2, drying perovskite that solidifies first around the carbon preventing further liquid infiltration; t3, a shrinkage cavity within the perovskite.

Figure 5A shows some examples of the infiltration defects and unwanted precipitates that can occur throughout the PSC stack. For instance, PbI_2_, a product created from the degradation of the perovskite material, appears in the form of bright crystals lodged between adjacent carbon flakes (Figure 5B) or near the top of the C layer where the reaction of the perovskite with moisture from the environment is exacerbated. Other inclusions resulting from contamination (Figure 5C) within the carbon stack are characterised by a high elemental concentration of silicon and oxygen and extremely low lead concentrations and can be associated with either retained polymer (contained in the screen-printing pastes) or contamination from the screen-printing process. These defects can be introduced due to the lack of a clean room environment when printing or via retained polymer from the ink and can include general fluff from wipes and clothing as well as organic components such as hair.

Large cavities were found within the carbon layer, as shown in Figure 5A,D, typically of the order of 0.5–2 microns in size. These can occur where adjacent or overlapping flakes prevent the downward flow of the wet perovskite precursor. It is also suspected that early crystallisation of the precursor occurs in the narrow gaps between carbon flakes, as illustrated in Figure 5 (t1–t3): the perovskite precursor infiltrates the cavity from the top and wets the inner walls, but its early crystallisation at the neck of adjacent flakes seals the top entry point before the cavity is fully infiltrated. This can be explained by rapid solvent burn-off owing to the high thermal conductivity of graphite materials. Whilst voids inside the carbon layer are undesirable, their dimensions are usually much smaller than the thickness of this layer, and their total volume is relatively low. Hence, they are unlikely to have an impact upon the device performance by causing major charge transfer resistance.

### 3.3. Infiltration Defects within the Active Layers

**Figure 6:**
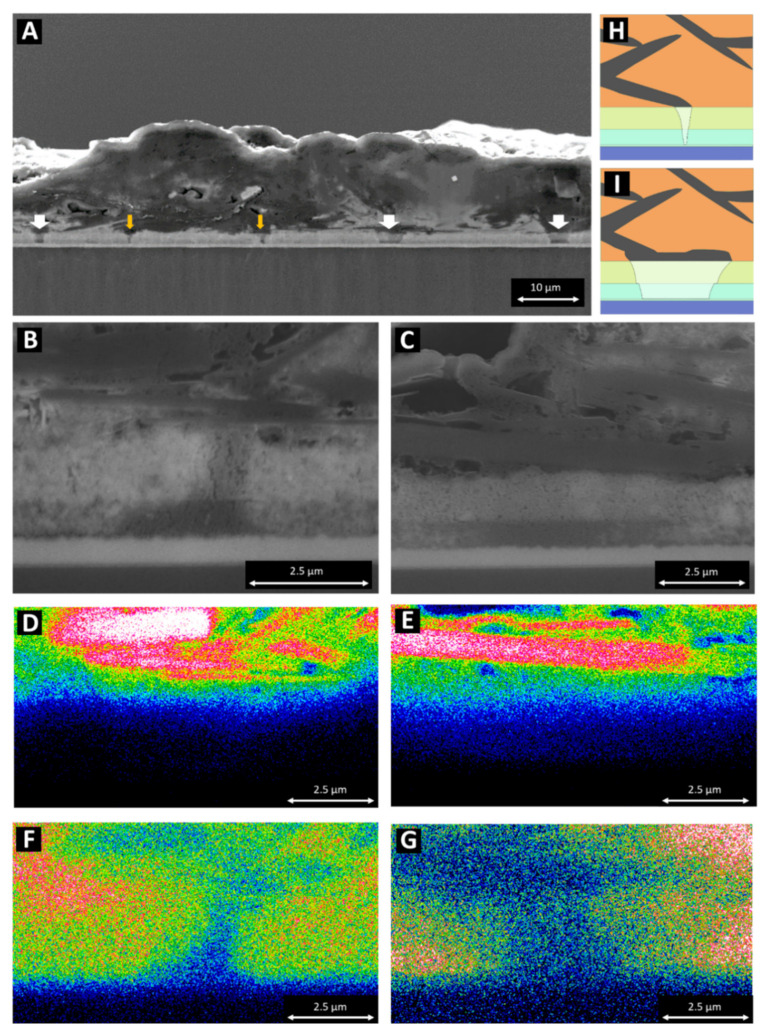
Imaging of pin and plate defects within the mesoporous carbon stack: (**A**) large cross section of a high-performance cell with plate (white arrows) and pin (orange arrows) defects identified; (**B**,**C**) close-up of a pin and a plate defect, respectively; (**D**,**E**) EDS carbon intensity map of a pin and a plate defect, respectively; (**F**,**G**) EDS lead intensity map of a pin and a plate defect, respectively; (**H**,**I**) simplified schematics of the pin and plate defects, respectively.

The large and variable dimensions of the carbon flakes, ranging from <1 μm to 20 μm, introduced great variability into the final structure and the overall thickness of the stack (Figure 6A). The thickness variation resulted in a change in the conductive properties of the overall layer, with the thinner areas presenting a lower area for current transfer. Large carbon flakes also tended to lie flat and pile up in agglomerates on top of the mesoporous stack of TiO_2_ and ZrO_2_, often blocking the perovskite infiltration underneath. This is particularly visible in Figure 5A, where perovskite voids are indicated in these layers. This type of defect is particularly detrimental to the performance of C-PSC devices since electrons cannot be readily extracted from the device in areas where the perovskite material is not in contact with the TiO_2_ mesoporous layer. Using correlative Raman mapping, the degradation of the perovskite material into lead iodide was previously shown to correlate with a loss in local device performance [17]; however, structural defects cannot be fully mapped using this technique, and other quality control tools will need to be developed in order to assess the yield and impact of these defects on a production line.

Typical carbon stacks with good performance do not show this fully collapsed blocking layer of carbon. Instead, individual horizontal graphite flakes in contact with the mesoporous ZrO_2_ introduce flow issues within the carbon stacks, as seen in Figure 6 In these images, two size defects can be seen, identified as pin (<2μm width) and sheet (>10μm width) defects. Pin defects are shown in Figure 6B,D,F, whilst plate defects are shown in Figure 6C,E,G. The pin defects were generally smaller and were located below a small lump of carbon or at the edge of a larger carbon flake. The morphologies and orientations of these smaller flakes or clumps could vary significantly, but it is believed that they were broken sections of larger flakes or locations where the flakes were angled such that some flow was still possible around them. This resulted in the small pinhole defects identified previously from a drop in activity (Figure 6B,H). Perovskite infiltration could also be inhibited by large agglomerations of carbon black. The larger sheet defects were typically isolated single sheets of graphite blocking the flow in their immediate area (as seen in Figure 6C,G). These defects were much larger, so they had a greater impact on the device performance, leaving large sections of the cell inactive.

Figure 6A shows an example of a relatively high-performing cell, with only two larger plate defects and two smaller pin defects, accounting for an active-area loss of 7%. In the images analysed in this study, cells with active areas of between 0% and 98% were observed, with a mean active area of 85%. The lower, middle, and upper quartiles were 62.7%, 85.65%, and 93.41%, respectively. This large range in performance indicates that this process is extremely irregular. All these devices were produced with the 5-AVAI additions identified by Filonik et al. [25], giving improved wettability and material backfilling. The variability shown in the filling of the mesoporous scaffold exhibited here occurred despite the improved infiltration and wettability of the 5-AVAI perovskites [17]. Early crystallisation of the perovskite prior to full infiltration, due to early heating on a hotplate, is likely to compound the issues with the carbon flakes described in this paper.

## 4. Conclusions

Mesoporous carbon stack solar cells infiltrated using a single-step process for CH_3_NH_3_PbI_3_ + AVAI were characterised using BBIM, to provide a new insight into the infiltration behaviours and the potential performance issues this may present in large-scale devices. The lack of optimisation for the screen printing of mesoporous carbon layers and the impact of flake orientation on the infiltration of MAPI-AVAI perovskite into the active layers were demonstrated. Pin and plate defects were related to the carbon flakes contacting the surface at the edge or in the flat area of the plate, respectively. The blocking effect of the flakes has been shown to affect, on average, 28% of the active area, with blocked areas ranging between 2% and 99%. A thorough understanding of these defects and the challenges they present in the large-scale production of C-PSCs devices will allow the development of improved printing processes, resulting in more consistent and higher-efficiency devices.

## Data Availability

Data sharing is not applicable.
